# *Vibrio vulnificus* sepsis after shrimp shelling in a patient with preexisting primary biliary cholangitis: a case report

**DOI:** 10.1186/s13256-023-03767-7

**Published:** 2023-01-28

**Authors:** Eishi Sakihara, Ikuma Noge, Hiroki Suzuyama, Hiroaki Takeoka, Shigeki Nabeshima

**Affiliations:** 1 General Medicine, Fukuoka University Hospital, 7-45-1 Nanakuma, Jonan-Ku, Fukuoka, 814-0180 Japan; 2 Tagawa Municipal Hospital, Tagawa City, Fukuoka Prefecture Japan

**Keywords:** *Vibrio vulnificus*, Primary biliary cholangitis, Shrimp shelling

## Abstract

**Background:**

*Vibrio vulnificus* is typically present in seawater, fish, and shellfish, and is known to cause severe sepsis, particularly in patients with liver diseases such as cirrhosis. *V. vulnificus* is one of the most dangerous waterborne pathogens, and infection mainly occurs in western Japan during the summer, with an increased fatality rate. Herein, we report the case of a patient with primary biliary cholangitis and sepsis caused by *V. vulnificus* infection sustained through shrimp shelling.

**Case presentation:**

An 82-year-old Japanese Asian woman with no medical history or underlying disease developed redness, swelling, and pain, which extended from the right fingers to the upper arm. A diagnosis of sepsis due to cellulitis was made. Blood culture detected *V. vulnificus*; thus, minocycline was administered in addition to meropenem. The disease course was uneventful, and the patient was discharged on day 28 of hospitalization. Symptoms in the right upper arm developed 1 day after the patient shelled a large number of shrimp; therefore, the infection route was assumed to be through wounds sustained during shrimp shelling. We suspected liver disease and measured serum anti-mitochondrial M2 antibody levels, leading to the diagnosis of primary biliary cholangitis.

**Conclusions:**

As in this case, small wounds caused by handling fish and shrimp are a potential source of infection. Patients with severe *V. vulnificus* infection should be thoroughly assessed for the presence of liver diseases such as primary biliary cholangitis.

## Background

The clinical and epidemiological characteristics of *Vibrio vulnificus* infection were first reported in 1979 by Blake *et al*. *V. vulnificus* is a slightly halophilic Gram-negative bacillus with an optimal salt concentration of 2–3% that lives in seawater and brackish water [1]. *V. vulnificus* infections mainly occur in western Japan during the summer, and it is considered one of the most dangerous waterborne pathogens, with a reported fatality rate of 50% [2]. Human infection occurs by eating raw or insufficiently heated food contaminated with *V. vulnificus*, exposure to seawater, or wounds inflicted by marine animals. *V. vulnificus* infection can result in fatal sepsis in patients with underlying diseases, such as preexisting liver disease, hemochromatosis, or immunodeficiency. To the best of our knowledge, there are no case reports of primary biliary cholangitis (PBC) associated with *V. vulnificus* infection. Herein, we report the case of a patient with PBC and sepsis caused by *V. vulnificus* infection sustained through shrimp shelling.

## Case presentation

The patient was an 82-year-old Japanese Asian woman with no comorbidities, relevant family medical history, alcohol consumption, or known allergies. No previous liver disease or abnormal liver function tests were noted. However, she had been a smoker of 20 cigarettes per day until 3 years prior. After mountain climbing on 23 and 24 July 2012, she experienced numbness and pain in the right arm. On 25 July, she developed general malaise. Since her symptoms did not improve by the morning of 26 July, she visited a private hospital with facial pallor, poor dietary intake, redness, mild swelling, and pain in the right arm. She was in circulatory shock with a blood pressure of 60/48 mmHg, and was later transported by an ambulance to Fukuoka University Hospital.

**Fig. 1:**
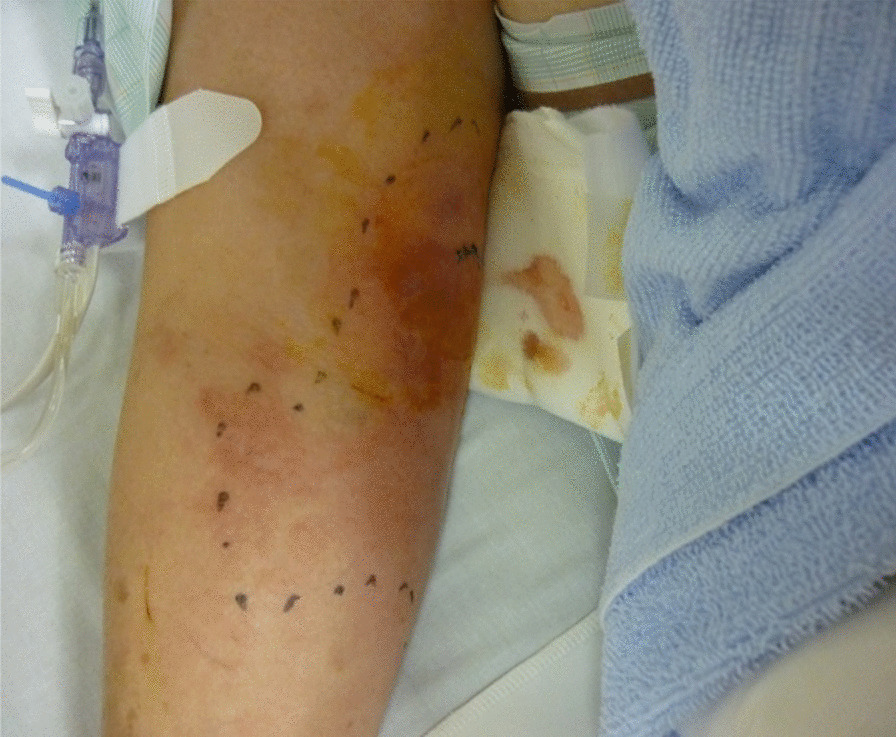
Skin rash in the right upper arm. Redness and swelling are observed above the right elbow on day 1. The area is warm to touch

**Fig. 2:**
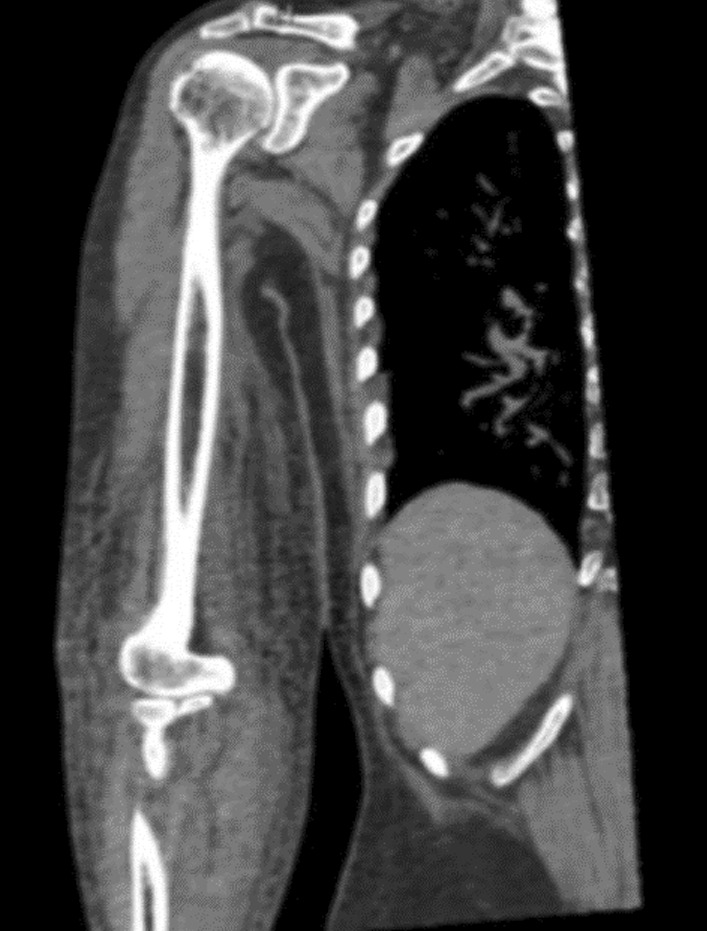
Computed tomographic examination of the right upper arm. A soft tissue opacity is seen in the right arm

Her physical findings on admission were as follows: body height, 158 cm; body weight, 51.0 kg; body mass index, 20.43 kg/m^2^; conscious and coherent; blood pressure, 66/33 mmHg; pulse rate, 91 beats per minute (bpm; regular); respiratory rate, 22 breaths/minute; SpO_2_, 97% (room air); body temperature, 37.7 °C; and redness and swelling extending from the medial part of the right upper arm to the right elbow and forearm (Fig. 1). Blood tests showed high levels of inflammation (Table 1), and computed tomography of the upper arm revealed adipose tissue turbidity (Fig. 2). We suspected septic shock, with the infection originating in the upper arm.

**Table 1:** Blood examination results on day 1

CBC	Numerical result	Biochemical	Numerical result		Numerical result
WBC	20,100/μL	TP	4.1 g/dL	K	2.7 mmol/L
Neutrophil	95.5%	Alb	1.9 g/dL	Cl	100 mmol/L
Lymphocyte	3.5%	BUN	21 mg/dL	HbA1c	5.2%
Monocyte	1.0%	Cr	1.1 mg/dL	ESR 1 hour	16 mm
Eosinophil	0.0%	T. bil	0.5 mg/dL	CRP	9.3 mg/dL
RBC	249 × 10^4^/μL	AST	44 U/L	Ferritin	2689 ng/mL
Hb	8.3 g/dL	ALT	24 U/L	Antinuclear Ab	< 40
Plt	9.2 × 10^4^/μL	ALP	118 U/L	RF	< 2 U/mL
Coagulation		γ-GTP	36 U/L	HBsAg	(–)
PT	17.6 seconds	LDH	183 U/L	HCV	(–)
PT-INR	1.57	Amy	< 20 U/L	Procalcitonin	50.74 ng/mL
FDP	8 μg/mL	Glu	118 mg/dL		
D-dimer	2.9 μg/mL	Na	133 mmol/L		

*CBC* complete blood count, *WBC* white blood cells, *RBC* red blood cells, *Hb* hemoglobin, *Plt* platelets, *PT* prothrombin time, *PT-INR* prothrombin time international normalized ratio, *FDP* fibrin degradation products, *TP* total protein, *Alb* albumin, *BUN* blood urea nitrogen, *Cr* creatinine, *T. bil* total bilirubin, *AST* aspartate aminotransferase, *ALT* alanine aminotransferase, *ALP* alkaline phosphatase, *γ-GTP* γ-glutamyl transpeptidase, *LDH* lactate dehydrogenase, *Amy* amylase, *Glu* glucose, *HbA1c* hemoglobin A1c (JDS), *ESR* erythrocyte sedimentation rate, *CRP* C-reactive protein, *RF* rheumatoid factor, *HBsAg* hepatitis B surface antigen

**Fig. 3:**
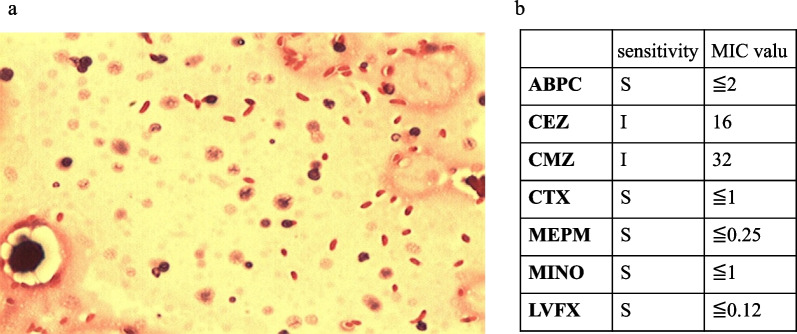
Bacteriological findings. **a** Blood culture Gram stain; **b** antibiogram of *V. vulnificus*. *ABPC* ampicillin, *CEZ* cefazolin, *CMZ* cefmetazole, *CTX* cefotaxime, *MEPM* meropenem, *MINO* minocycline, *LVFX* levofloxacin

First, to stabilize blood pressure, we rapidly administered 1000 mL of lactated Ringer’s solution, an initial dose of meropenem 1.0 g after blood culture specimens were collected, and noradrenaline at 0.2 µg/kg/minute. Then, under local anesthesia, a 5-cm incision was made distally from the medial right upper arm and proximally from the medial right cubital fossa. The color tones of the subcutaneous fat and fascia were normal, and we suspected cellulitis rather than necrotizing fasciitis. For the septic shock, we continued to administer meropenem (3.0 g/day) for 12 days, with linezolid (1.2 g/day) for 5 days and immunoglobulin (5 g/day) for 3 days via intravenous injection. On day 2 of hospitalization, a blood culture detected Gram-negative bacilli (Fig. 3a), and linezolid was discontinued. On day 5 of hospitalization, *V. vulnificus* was identified by blood culture, and we added minocycline (100 mg) twice daily for 8 days, to which *V. vulnificus* was susceptible (Fig. 3b). Subsequently, the inflammatory findings rapidly improved; the patient’s C-reactive protein levels dropped from 9.3 to 1.1 mg/dL in 9 days. Although there was no history of seafood intake or contact with seawater, we learned that the swelling developed in the right hand 1 day after she had shelled a large number of raw shrimp for cooking, and the swelling spread to the trunk. Therefore, we believed that the route of *V. vulnificus* infection was through minor skin wounds inflicted by shrimp shells.

**Fig. 4:**
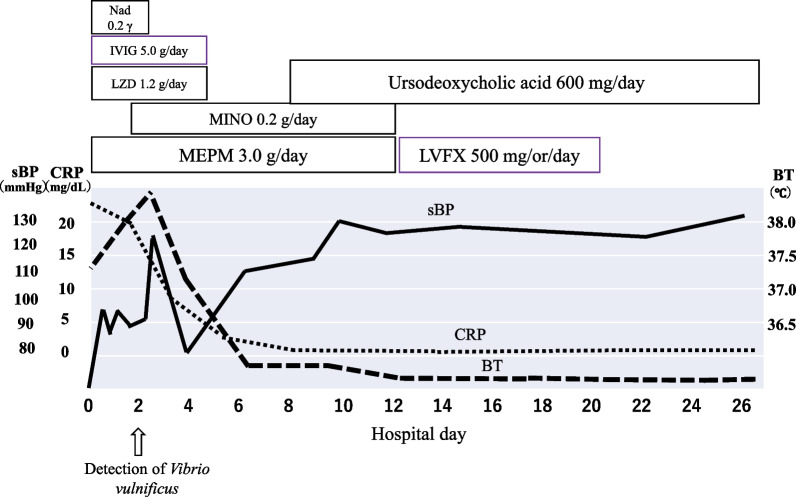
Disease course after admission. *Nad* noradrenaline, *IVIG* intravenous immunoglobulin, *LZD* linezolid

On day 11 of hospitalization, the treatment was switched to oral levofloxacin (500 mg daily), as the redness and swelling in the upper arm had improved. After 7 days, drug-induced thrombocytopenia was observed, and levofloxacin was discontinued. No recurrence was observed, and the patient was discharged on day 28 of hospitalization (Fig. 4).

During the course of hospitalization, persistent elevation of serum γ-glutamyl transpeptidase, alkaline phosphatase, aspartate aminotransferase, and alanine aminotransferase levels suggested liver disease. Blood tests performed on day 5 of hospitalization showed positivity for anti-mitochondrial M2 antibodies (Table 2), and abdominal echography showed a pattern of chronic liver disease, leading to the diagnosis of PBC. Since the liver damage was mild, we started to administer ursodeoxycholic acid (600 mg/day). On follow-up with the practitioner, we received no reports of exacerbations.

**Table 2:** Blood examination results on day 5

CBC	Numerical result	Biochemical	Numerical result
WBC	3400/μL	T. bil	0.3 mg/dL
Neutrophil	76.7%	AST	39 U/L
Lymphocyte	12.2%	ALT	32 U/L
Monocyte	5.7%	γ-GTP	108 U/L
Eosinophil	5.1%	LDH	112 U/L
Hb	9.0 g/dL	Amy	35 U/L
Plt	6.4 × 10^4^/μL	Anti-mitochondrial M2 Ab	167 U/mL

*CBC* complete blood count, *WBC* white blood cells, *Hb* hemoglobin, *Plt* platelets, *T. bil* total bilirubin, *AST* aspartate aminotransferase, *ALT* alanine aminotransferase, *γ-GTP* γ-glutamyl transpeptidase, *LDH* lactate dehydrogenase, *Amy* amylase

## Discussion and conclusions

In the present case, the cause of sepsis was initially unclear because the patient had no relevant medical history or underlying disease. However, blood culture revealed a causative organism, *V. vulnificus*, and the history of shrimp shelling was identified. We thought the fungus entered through a small wound and became the source of infection. Moreover, *V. vulnificus* infection tends to accompany liver disease, and persistent elevation of serum γ-glutamyl transpeptidase, alkaline phosphatase, aspartate aminotransferase, and alanine aminotransferase levels suggested liver disease, diagnosed as PBC in our patient. This is an interesting case study because we could not find any similar case reports.

*V. vulnificus* is a Gram-negative bacillus that causes wound infections and sepsis in some patients. The outcomes of *V. vulnificus* infection tend to be serious in men, and in middle-aged and older patients (> 40 years of age), particularly those with underlying diseases, such as liver disease, diabetes, immune disorders, elevated serum iron concentration, and cirrhosis (mainly alcohol induced) [2]. *V. vulnificus* infection is characterized by a short incubation period, typically within 24 hours of exposure. Moreover, since this bacterium can cause severe infections, such as bacteremia and wound infections, prompt initiation of antibacterial therapy is essential. Therefore, if an infection is suspected, particularly in high-risk patients, it is crucial to identify this bacterium accurately and rapidly in clinical practice [2]. In one report, regarding the rates of sepsis and wound infection, *V. vulnificus* caused wound infections and bacteremia in 62 patients over the course of 1 year in Israel. Among them, 57 patients developed cellulitis, four developed necrotizing fasciitis, and one developed osteomyelitis. The fatality rate of *V. vulnificus* infection has been reported to be 20–50%, and no deaths were observed in this particular report [1].

The proliferation of *V. vulnificus* is influenced by water temperature. *V. vulnificus* accounted for approximately 8% of the aerobic bacteria in samples collected from the Chesapeake Bay between April 1991 and December 1992. It was not detected in February or March (water temperature < 8 °C) but was detected in 80% of the samples in May, July, September, and December (water temperature > 8 °C) [3]. Regarding the salt concentration of the water, when the influx of freshwater from the Mississippi River reduced the salt concentration at the Mississippi shoreline, *V. vulnificus* became temporarily undetectable in this area. Therefore, salt concentration greatly influences the growth of *V. vulnificus* [4]. In the present case, although the location at which the shrimp were caught was unknown, the disease onset in July did not contradict the biology of *V. vulnificus*.

PBC, which is an autoimmune disease associated with environmental and genetic factors, was first reported by Addison *et al.* in 1857; however, its exact cause remains unknown [5]. A study of 1032 patients with PBC revealed that it is more common in women and is sometimes accompanied by other autoimmune diseases such as Sjögren’s syndrome and Raynaud syndrome [6]. A family history of PBC, smoking, and history of urinary tract infections are also commonly reported. Furthermore, it has been suggested that in urinary tract infections, *Escherichia coli* can disrupt immune tolerance, leading to the development of PBC [6, 7]. In the present case, PBC was revealed by blood test on the fifth day of onset. It is possible that *V. vulnificus* infection occurred after the patient originally had PBC, or that PBC developed after *V. vulnificus* infection. It is also possible that the two diseases coincidentally overlap. However, there are no precedents for either of these possibilities, and we cannot discuss these possibilities at this time.

In conclusion, the lessons learned from this case suggest that it is useful to evaluate patients with *V. vulnificus* infection for the presence of undiagnosed liver disease, such as asymptomatic PBC. In addition, a detailed history regarding *V. vulnificus* infection should be obtained because shelling of crustaceans is a potential source of infection.

## Data Availability

Not applicable.

## Abbreviation

PBC: Primary biliary cholangitis
